# CD4-Specific Designed Ankyrin Repeat Proteins Are Novel Potent HIV Entry Inhibitors with Unique Characteristics

**DOI:** 10.1371/journal.ppat.1000109

**Published:** 2008-07-25

**Authors:** Andreas Schweizer, Peter Rusert, Livia Berlinger, Claudia R. Ruprecht, Axel Mann, Stéphanie Corthésy, Stuart G. Turville, Meropi Aravantinou, Marek Fischer, Melissa Robbiani, Patrick Amstutz, Alexandra Trkola

**Affiliations:** 1 Division of Infectious Diseases, University Hospital Zurich, Zurich, Switzerland; 2 Center for Biomedical Research, Population Council, New York, New York, United States of America; 3 Molecular Partners AG, Zurich-Schlieren, Switzerland; Harvard Medical School, United States of America

## Abstract

Here, we describe the generation of a novel type of HIV entry inhibitor using the recently developed Designed Ankyrin Repeat Protein (DARPin) technology. DARPin proteins specific for human CD4 were selected from a DARPin DNA library using ribosome display. Selected pool members interacted specifically with CD4 and competed with gp120 for binding to CD4. DARPin proteins derived in the initial selection series inhibited HIV in a dose-dependent manner, but showed a relatively high variability in their capacity to block replication of patient isolates on primary CD4 T cells. In consequence, a second series of CD4-specific DARPins with improved affinity for CD4 was generated. These 2nd series DARPins potently inhibit infection of genetically divergent (subtype B and C) HIV isolates in the low nanomolar range, independent of coreceptor usage. Importantly, the actions of the CD4 binding DARPins were highly specific: no effect on cell viability or activation, CD4 memory cell function, or interference with CD4-independent virus entry was observed. These novel CD4 targeting molecules described here combine the unique characteristics of DARPins—high physical stability, specificity and low production costs—with the capacity to potently block HIV entry, rendering them promising candidates for microbicide development.

## Author Summary

There is an increasing need to develop inhibitors of HIV entry into target cells for both application in therapy and prevention. The development of specific HIV inhibitors as microbicides, agents that by topical application prevent infection, is considered particularly important in limiting the spread of HIV in the absence of effective vaccines. To derive highly potent and specific inhibitors of HIV entry for potential use as microbicide, we employed the recently developed Designed Ankyrin Repeat Protein technology. Using this technique, Designed Ankyrin Repeat Proteins can be evolved that bind their target molecules as specifically and efficiently as antibodies. In the present study, we generated a panel of Designed Ankyrin Repeat Proteins that bind specifically to the cellular CD4 receptor, the main entry receptor of HIV. The obtained proteins are very potent and highly specific inhibitors of HIV entry and provide a broad reactivity against genetically different virus strains. Due to the high physical stability of Designed Ankyrin Repeat Proteins and their low cost production, these novel HIV entry inhibitors represent promising candidates for microbicide development.

## Introduction

The increasing need for a vaccine to control the HIV pandemic is undoubted, but recent failures of vaccine programs have made clear that it will be years to decades before a successful vaccination program can be installed [1]. In the meantime, drug based intervention strategies must be found to fill the gap and put the continuous spread of HIV at halt, particularly in resource poor settings where 90% of the estimated 33 million HIV infected individuals live [2].

HIV infection is predominantly acquired via heterosexual transmission across mucosal surfaces [3]. Strategies that prevent mucosal transmission are therefore considered to significantly impact on diminishing viral spread [4]. Microbicides, agents that by topical application on mucosal surfaces protect from HIV infection, are regarded as one of the most promising preventive intervention strategies in the absence of effective vaccination programs [2],[4],[5]. The sought for microbicides against HIV have to fulfill highly specific requirements: Besides promoting strong and reliable protection from HIV infection, these compounds have to be inexpensive, readily available, stable, well tolerated and easy to apply to allow a wide spread use. Recent efforts in microbicide research have mainly focused on chemical compounds of relatively simple composition that provide protection from HIV infection by largely nonspecific (non HIV specific) mechanisms as for instance charge-charge interactions [6]. Although *in vivo* efficacy of two such candidate microbicides, nonoxynol-9 [7] and cellulose sulfate [8], could not be established [9]–[12] several other pan-reactive molecules are in development that show promise [4],[6],[13]. As for all drug interventions against HIV, combination therapy will likely also be necessary in microbicide application to reach potent and broad efficacy. Thus microbicides that target HIV specifically and potentially can be used in combination with pan-reactive molecules are urgently sought for.

Prime targets for microbicide attack are the virus and cellular proteins involved in the early events in infection: the entry receptors CD4, CCR5 and CXCR4, the viral envelope proteins and compounds that interfere post entry with reverse transcription and integration of HIV into the host cell. Application of specific HIV inhibitors targeting these events as topical microbicides has proven effective in blocking mucosal HIV transmission in the SHIV macaque infection model underlining their potential in HIV prevention [4], [14]–[18].

To date only few small molecules that inhibit HIV entry have been defined [4]. While protein-based inhibitors are commonly more expensive in production, they can have functional advantages. Most importantly, they provide outstanding target specificity since the contact area between agent and target protein is formed by comparatively large surface patches as for instance in antibody-antigen interactions.

The aim of our study was to derive inhibitors of HIV entry that achieve the desired specificity and potency together with the high physical stability and low production costs required for the application as microbicide. To this end, we made use of the recently established Designed Ankyrin Repeat Protein (DARPin) technology which is based on the principle of naturally occurring ankyrin repeat proteins, a ubiquitously expressed family of proteins mediating specific protein-protein interactions across species [19]. DARPins were designed as an alternative to antibodies: they share the antibodies' ability to be selected and to bind any given target with high affinity and specificity but are clearly superior in terms of physical stability and production costs [20],[21]. Highly diverse DARPin DNA libraries, comprising at least 10^11^ different sequences per reaction, have successfully been employed to identify enzyme inhibitors and specific binding proteins in diverse biological systems [22]–[29].

The specificity and high affinity achieved in DARPin-target interactions, paired with the fact that the 12 to 19 kDa DARPin proteins have a remarkable physical stability and are expressed in prokaryotic systems, allowing large scale production at relatively low costs, renders DARPins promising candidates for the selection of HIV inhibitors. Here, we report the successful selection and characterization of CD4-specific DARPins and their function as broadly active inhibitors of HIV entry, which underlines the potential of this novel type of inhibitor molecules in HIV infection.

## Methods

### Ribosome display and selection for binders with improved affinities

An introduction into the DARPin technology and ribosome display is provided as Supporting Information (Protocol S1 and Figures S1 and S2). Detailed specifics on the use and generation of DARPin libraries has been described previously [20]. Here, N2C and N3C libraries encoding for DARPins consisting of an N- and a C-terminal capping repeat, and either two (N2C) or three (N3C) internal ankyrin repeat modules containing randomized amino acid residues, were used. The theoretical diversity of the N3C library is 3.8×10^23^. Ligated library DNA used in the selection described here encoded for a minimum of 10^11^ individual members [20]. The diversity of the library is further increased by introducing errors through the polymerase used in subsequent PCR cycles. Library selections were performed against the tetrameric CD4 fusion protein CD4-IgG_2_ (kindly provided by Bill Olson, Progenics Pharmaceuticals; [30]) which was immobilized via a Fab-specific anti human IgG-antibody (Sigma). For selections, PCR-amplified libraries were transcribed and three standard ribosome-display selection rounds were performed as described [23],[31],[32]. Two alternate approaches were probed in the fourth selection round to achieve highly specific binders: i) a standard ribosome display selection round with more extensive washing (3 h in total) and ii) the use of purified gp120 of the R5-tropic virus JR-FL (1 µM; kindly provided by W. Olson Progenics Pharmaceuticals) to elute binders that compete with viral glycoprotein for binding to CD4. The RT-PCR products of the genes obtained after both fourth cycles were combined in a pool (termed 1^st^ series binders) and then used for a single clone analysis as described below.

In a separate line of experiments we aimed to select binders with improved affinities for CD4. To this end, all round 3 and round 4 sublibraries were transcribed and translated *in vitro* as described [33]. Then the ternary complexes of ribosome, mRNA, and displayed proteins were equilibrated with 1 nM biotinylated CD4-IgG_2_ at 4°C for 1 h before 1 µM non-biotinylated CD4-IgG_2_ was added. The aliquots were incubated for 3 h at 4°C and the complexes were recovered by binding to streptavidin-coated magnetic beads (Roche Applied Science) for 30 min. The beads were washed five times, and the RNA was eluted and purified as described [33]. The pool of binders derived from this affinity selection was termed 2^nd^ series binders and characterized as described below.

### Detection of selected binders by ELISA

CD4-IgG_2_ was immobilized via a Fab-specific anti-IgG capture antibody (Sigma) on Maxisorp 96-well plates (Nunc). To screen for CD4 binders, 100 µl each of crude *Escherichia coli* extracts containing DARPins or purified DARPins were applied to wells containing immobilized CD4-IgG_2_ and to wells containing the capture antibody alone. Bound DARPins were detected upon incubation with anti-RGS-His antibody (Qiagen), anti-mouse-IgG-alkaline phosphatase conjugate (Sigma) and p-nitrophenylphosphate (Sigma) as substrate. Wells without CD4-IgG_2_ were used as negative controls to verify the binding specificity of the tested DARPins.

### Competition ELISA

For the gp120 competition ELISA the same setup as described above was employed. CD4-IgG_2_ coated plates were incubated with JR-FL gp120 (0–800 nM; kindly provided by Progenics Pharmaceuticals) for 1 h at 25°C before pure DARPins (200 nM) were added. Detection and readout was carried out as described above.

For the competition ELISA using CD4-directed monoclonal antibodies (mAbs) as competitors, soluble CD4 (20 nM, Progenics Pharmaceuticals) was biotinylated using EZ-link sulfo-NHS-LC-biotin (Pierce) according to the manufacturer's instructions and immobilized via neutravidin (Pierce, 66 nM) on Maxisorp 96-well plates (Nunc). mAbs L222, Q4120, 13B82 [34],[35] and 5A8 [36] were kindly provided by Q. Sattentau. DARPin (20 nM) plus different CD4-antibodies (66 nM) were added and incubated at 25°C for 1 h. Bound DARPins were detected by ELISA using an anti-poly-His-alkaline phosphatase conjugate (Sigma) as described above. Wells without added antibody where included as control and defined as 0% competition. Competition was rated as follows: −, +, ++, and +++ for signal decreases of 0–25%, 25–50%, 50–75% and 75–100%, respectively.

### Protein purification and endotoxin removal

DARPins were produced in soluble form in *E. coli* and purified using Ni-NTA affinity chromatography as described [37]. Endotoxins (lipopolysaccharides) were removed using 0.1% Triton X-114 as described [38] and the DARPins were further purified using EndoTrap red columns (Profos) according to the manufacturer's recommendations. The remaining endotoxin content was determined using the kinetic chromogenic limulus amebocyte lysate assay (Endotell) according to the manufacturer's instructions. All DARPin preparations used for investigation of cellular activation had endotoxin levels below 0.5 EU/mg.

### Surface plasmon resonance (SPR)

All SPR measurements were performed at 25°C using a Biacore 3000 instrument and a SA sensor chip (Biacore). To immobilize CD4-IgG_2_, the protein was first chemically biotinylated using EZ-Link sulfo-NHS-LC-biotin (Pierce). The individual DARPins were applied in various concentrations (0.25–1'000 nM, depending on affinity) to a flow-cell with immobilized CD4-IgG_2_ for 180 s at 50 µl/min, followed by washing with buffer. The signal of an uncoated reference cell was subtracted from the measurements. The kinetic data of the interactions were evaluated with a global fit using the BIAevaluation 3.0 software (Biacore).

### Generation of human mouse CD4 domain 1 chimera

A chimeric construct coding for human CD4, where the human domain 1 sequence is replaced by its murine homologue sequence, was constructed as follows: in pEYFP-N1-hCD4 (a kind gift from Jun-ichi Fujisawa [39]), an expression vector for human CD4, a ScaI restriction site was introduced at position 10 in CD4-domain 1 by two conservative nucleotide exchanges via site directed mutagenesis (QuikChange XL, Stratagene), resulting in plasmid pEYFP-N1-hcD4-Sca. The murine CD4-D1 domain was amplified by PCR from the plasmid pCMV-Sport6-mCD4 with primers mD1_fw: gtcactcaagggaagacgctagtactggggaaggaaggg and mD1_rev: ggtcaggctctgcccctgcagcaggtgggtacccggactgaagg. The PCR product and pEYFP-N1-hCD4-Sca, which harbour unique ScaI and AarI restriction sites, were digested with these two enzymes and the PCR-derived insert encoding the murine CD4-domain 1 was ligated into the human CD4 plasmid finally resulting in pEYFP-N1-hCD4mD1.

### Immunofluorescent staining and analysis

Cells (100'000/well) were incubated with DARPins (200 nM) for 20 min at 25°C. Bound DARPin was detected using anti-RGS-His antibody (Qiagen) and goat-anti-mouse phycoerythrin labeled antibody (Caltag). Binding of DARPins to CD4^+^ A3.01 cells, CD4^−^ A2.01 cells (NIH AIDS Research & Reference Reagent Program, No. 2059 and 166), CEM5.25luc.gfp (CD4^+^; provided by N. Landau) and TZM-bl cells (CD4^+^; [40]) was investigated. Cells were washed three times between all incubation steps using PBS containing 0.1% azide and 1% BSA. After the last step, cells were fixed (in PBS, 0.1% azide, 1.25% formaldehyde) and subjected to flow cytometry using a FACSCalibur flow cytometer (BD Biosciences) and Flowjo software (Tree Star).

To measure the effect of DARPin on cellular CD4 expression, untouched CD4^+^ T cells were isolated from CD8-depleted peripheral blood mononuclear cells (PBMC) of healthy donors using the CD4^+^ T cell isolation kit II (Miltenyi Biotech) according to the manufacturer's instructions. Purity of the isolated CD4^+^ T cells was routinely >97%. CD4^+^ T cells were cultured in the presence or absence of the indicated DARPins at 200 nM for 1 h, 3 h, or 18 h. Thereafter, CD4^+^ T cells were washed twice, stained with PE-labeled anti-CD4 (Caltag) and analyzed for CD4 expression by flow cytometry.

To analyze overlapping binding patterns amongst the selected CD4 specific DARPins, competition of DARPins to bind to cellular CD4 was investigated. To this end, DARPins 29.2 and 57.2 were chemically modified with the HLX_633_ fluorescent dye (Invitrogen) according to the manufacturer's recommendations and purified by size exclusion using NAP5 columns (GE Healthcare). CD4^+^ A3.01 cells were incubated with the fluorescently labeled DARPins at 20 nM (10′, 25°C) followed by addition of the unlabeled DARPins (1 µM, 20′, 25°C). Cells were washed thereafter and analyzed by flow cytometry.

To define the domain-specificity of selected DARPins, 293T cells were transiently transfected with plasmids pEYFP-N1-hCD4 (see above), pCMV-Sport6-mCD4 (obtained from RZPD) or the newly created chimeric construct pEYFP-N1-hCD4mD1 (see above) with 25 kD polyethylenimine (Polysciences) as described [41] and stained 48 h post transfection with fluorescently labeled DARPins at a concentration of 5 to 50 nM and subsequently analyzed by flow cytomtery.

### Stimulated primary CD8-depleted PBMC

CD8^+^ T-cell depleted (Rosette Sep cocktail, StemCell Technologies Inc.) PBMC were isolated by Ficoll-Hypaque centrifugation of buffy coats obtained from three healthy blood donors. Cells were adjusted to 4×10^6^/ml in culture medium (RPMI 1640 medium, 10% fetal calf serum, 10 U/ml interleukin-2, glutamine, and antibiotics), divided into three parts, and stimulated with 5 µg/ml phytohemagglutinin (Sigma), 0.5 µg/ml phytohemagglutinin, or anti-CD3 mAb OKT3 as previously described [42]. After 72 h, cells from all three stimulations were combined (referred to as three-way-stimulated PBMC) and used as a source of stimulated CD4^+^ T cells for infection and virus isolation experiments.

### Replication competent viruses

Replication competent viruses were produced by infection of three-way stimulated PBMC. The 50% tissue culture infectious dose (TCID_50_) was determined by end point dilution. Infections were detected by p24 ELISA. In sum 10 subtype B viruses, including 7 R5 users (JR-FL, SF-162, Pat 17, Pat 020, Pat 111, Pat 114, Pat 120) and 3 X4 users (NL4-3, 2044 and Pat 19) were probed. Pat 17 is a R5 tropic primary isolate derived from plasma of a chronically HIV infected individual as described [43]. The origin of the other viruses has been described previously [42],[43].

### Env-pseudotyped HIV

Env-pseudotyped HIV was generated by transfection of 293T cells with plasmids encoding the reporter gene expressing virus backbone, pNLluc [44] (kindly provided by A. Marozsan and J. P. Moore) and the respective functional envelope clone using 25 kD polyethylenimine as described [41]. Viral supernatants were harvested 2 days post transfection and the TCID_50_ was determined by end point dilution. Infections were measured by firefly luciferase activity (Bright-Glo Luciferase Assay System, Promega). Plasmids encoding envelopes of R5 using viruses of subtype B (AC10.0.29, PVO.4, QHO692.42, REJO4541.67, RHPA4259.7, SC422661.8, TRJO4551.58, TRO.11, WITO4160.33) and subtype C (DU123.4, DU151.2, DU156.2, DU422.1) were kindly provided by D. Montefiori [45],[46]. Plasmids encoding envelopes of JR-FL and NL4-3 were provided by N. Landau and the plasmid encoding the envelope of SF162 was provided by L. Stamatatos.

### Neutralization assays using *env*-pseudotyped virions on TZM-bl cells

Neutralization assays on TZM-bl cells using pseudotype viruses were performed as described [40]. Briefly, TZM-bl cells (10'000/well; 96well format) were preincubated for 1 h at 37°C with serial dilutions of DARPins and were then infected with aliquots of the viruses (100 TCID_50_) together with DEAE dextran (10 µg/ml) in a total infection volume of 200 µl. After three days, the cells were lysed using Glo lysis buffer (Promega) and luciferase activity determined upon addition of Glo substrate (Promega) on a Dynex Technologies Luminometer. The DARPin concentration causing 50, 70, 90% reduction in luciferase reporter gene production after 48 h was determined by regression analysis.

Potential synergistic effects of combinations of the CD4-specific DARPin 25.2 with other entry inhibitors were investigated with JR-FL pseudotyped virus on the TZM-bl reporter cell line. Combination indices [CI] were calculated using the Loewe additivity formula [47]–[49]:
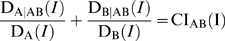
D_A_ *(I)* is the dose of drug A alone required to result in inhibition *I* and D_A|AB_ *(I)* the dose of drug A in the combination of A+B required to give the inhibition *I*. CI of 1 indicates additivity, <1 synergy and >1 antagonism.

### PBMC based neutralization assay

Inhibition of replication-competent virus infection of primary human CD4 T cells was assessed essentially as described [50]. Briefly, stimulated CD8 depleted PBMC (100'000/well) were preincubated for 1 h with DARPins at 37°C, followed by infection with the respective replication-competent virus (100 TCID_50_). After incubation for 6 to 8 days, p24 antigen production was determined in cellular supernatant by ELISA as described [49],[51]. The DARPin concentration causing 70% reduction in p24 antigen production was determined by regression analysis as described [42].

For macaque PBMC based neutralization assays, macaque PBMC were cultured with 5 µg/ml of PHA-P (Sigma) for 3 days, before being plated at 2×10^5^ cells per well of a 96 well plate (Becton Dickinson) in medium with 50 U/ml IL-2. Graded doses of the CD4-specific DARPin 25.2 or the control E3_5 DARPin were added to each well (duplicates per dose) and incubated for 1 h at 37°C. After the incubation, 1000 TCID_50_ of SIVmac239 was added to each well (with 50 U/ml IL-2). The cells were cultured for 7 days (adding more IL-2 every other day), after which the cells were collected and lysed for PCR. SIV infection was measured using a Q-PCR assay for SIV gag DNA [52],[53]. The DARPin concentration causing 90% reduction in SIV gag DNA was determined by regression analysis.

### Effects of DARPins on dendritic cells (DC)

Monocytes were isolated from PBMC by positive selection with CD14 microbeads (Miltenyi). Purified monocytes were cultured for 4 days in RPMI-10% FCS containing 1000 U/ml GM-CSF and 1'000 U/ml IL-4 (both from Immunotools). Monocyte-derived DC were then washed twice, seeded at 1×10^6^/ml and treated with the purified DARPin preparations (375 nM) for 24 h. *E. coli* lipopolysaccharide (2.5 EU/ml; Charles River Endosafe) was used as control. Finally, to assess the activation status of the cells, DC were stained with PE-labeled anti-CD80 (BD Biosciences) and with propidium iodide (BD PharMingen) and CD80 expression levels were quantified by flow cytometry.

### Assessment of T cell proliferation

Labeling of PBMC with CFSE (carboxy-fluorescein succinimidyl ester) was performed as described [54]. Briefly, CD8-depleted PBMC from a single donor were stained 8 min at room temperature with 3 µM CFSE (Molecular Probes). Staining was stopped by addition of an equal amount of FCS and cells washed three times with PBS containing 1% FCS. CFSE-labeled cells were incubated with 500 nM endotoxin purified DARPin (1 h at 37°C) and cultured for 4 days in RPMI 1640 containing 10% FCS, antibiotics, 100 U/ml interleukin-2 and anti-CD3 mAb OKT3. The cells were analyzed by flow cytometry using anti-CD3-PE and propidium iodide for gating. Proliferation of cells was assessed on the basis of the shifts in the CFSE- labeling intensity using the FlowJo software as described [54].

### Assessment of T helper memory cell function in presence of CD4 specific DARPin

To assess whether CD4-specific DARPins interfere with T helper memory cell functions, activation of antigen-specific T cells in presence and absence of DARPin 55.2 and the control E3_5 using a standard interferon-γ ELISpot assay was assessed [55]. Briefly, 96-well membrane plates (MAIP S45, Millipore) were coated overnight with anti-human IFN-γ antibody (1-D1K, MAbtech). CD8-depleted PBMC were isolated one day prior to the experiment and cultured in RPMI 1640 containing 10% FCS and antibiotics overnight. The next day cells were preincubated with 200 nM (streptokinase/streptodornase experiment) or 250 nM (cytomegalovirus experiment) endotoxin free DARPins 55.2 and E3_5 for 1 h at 37°C. Cells (2×10^5^) were then seeded into wells of the coated 96-well membrane plates and stimulated with either streptokinase/streptodornase (400 U/ml) or cytomegalovirus (CMV)-lysate (10 µg/ml) overnight at 37°C. Phytohaemagglutinin (10 µg/ml) was used as positive control. IFN-γ production was detected by sequential addition of a detection antibody cocktail containing a biotinylated anti-human IFN-γ antibody (7-B6-1, MAbtech), streptavidin alkaline phosphatase (MAbtech), followed by washing. AP (alkaline phosphatase) conjugate substrate kit (Biorad) was used and the resulting colored spots were quantified using an ELISpot reader (AID). Background reactivity observed in cultures without stimulation was subtracted and results are expressed as specific spot forming cells (SFC) per 10^6^ CD8-depleted PBMC.

### Interference of DARPins with CD4:MHC class II interaction

To study if CD4 specific DARPins interfere with CD4:MHC class II interaction directly, we performed a cell based binding assay based on rosette formation between CD4 and MHC class II expressing cells [56]. Briefly COS-7 cells (ATTC CRL-1651; cultivated in DMEM, 10% FCS) were seeded at a density of 200'000 cells per 6-well, and one day later transfected with the CD4 encoding plasmid pEYFP-N1-hCD4 ([39]) using the Ca-phosphate transfection system (Promega) according to the manufacturer's instructions. Transfection medium was replaced 8 h later and two days post transfection cells were utilized in the rosette assay. To this end CD4 expressing and control COS-7 cells were treated with CD4 specific DARPins (23.2, 25.2, 27.2, 29.2, 55.2, 57.2), and a control DARPin (E3_5), buffer or the anti-CD4 antibody Q4120 specific for domain 1 (Sigma; 100 nM), which is known to block CD4 binding to MHC II, for 30 min at 37°C at a concentration of 50 nM or 200 nM. Subsequently, medium was removed, and cells incubated with 1×10^7^/well Raji B cells (NIH AIDS Research & Reference Reagent Program, No. 9944) cultivated in RPMI1640, 10% FCS) containing identical concentrations of inhibitors. After 1 h incubation at 37°C non-adherent cells were removed by washing the wells gently seven times with medium. Cells were then fixed with 1.5% paraformaldehyde (PFA) and rosette formation assessed microscopically.

### Crossreactivity with macaque CD4

Crossreactivity with rhesus CD4 was investigated using PBMC from adult male and female chinese rhesus macaques (*Macaca mulatta*) which were housed at the Tulane National Primate Research Center (TNPRC; Covington, USA). Animals were anesthetized with ketamine-HCl (10 mg/kg) prior to heparinized blood samples being taken (no more than 10 ml/kg/month/animal). Protocols were reviewed and approved by the Institutional Animal Care and Use Committee of the TNPRC. Animal care procedures were in compliance with the regulations detailed in the Animal Welfare Act and in the “Guide for the Care and Use of Laboratory Animals”. PBMC were isolated using Ficoll-Hypaque density gradient centrifugation (GE Healthcare). Cells were washed twice in 1× PBS and resuspended in FACS wash (FW) buffer (1× PBS supplemented with 1% human serum and 1 mM EDTA, both from Sigma). For DARPin staining, 4×10^5^ macaque PBMC were resuspended in 50 µl FW buffer in a 96 well plate (BD Biosciences). DARPins, 2 µl of each (5 µM), were added to the cells and incubated for 20 min at 4°C. Cells were washed twice in FW buffer and CD4 T cells were identified using a 1/25 dilution of FITC-conjugated anti-CD3 (clone Sp34, BD PharMingen) and PE-conjugated anti-CD4 (clone L200, BD PharMingen). PE- and FITC-conjugated isotype Ig controls were included in all experiments and typically gave signals <1 log of fluorescence. To detect DARPin binding, cells were incubated with a 1/100 dilution of the anti-Penta-His Alexa Fluor 647 conjugate (Qiagen). The DARPin negative control was no DARPin with anti-Penta-His Alexa Fluor 647. Gates were set to include all mononuclear leukocytes based on the forward- and side-scatter characteristics (excluding any contaminating neutrophils). The gates used to define the CD3/CD4 cells were determined based on the isotype controls. All samples were acquired on a FACSCalibur (BD Biosciences) and analyzed using FlowJo software (Tree Star, Inc). Mean fluorescent intensities (MFI) of DARPin staining in the CD3/CD4 population were adjusted by subtracting the MFI of the negative DARPin control. Standard deviations represent n = 4 animals, processed and stained in parallel.

## Results

### Selection and biochemical characterization of CD4-specific DARPins

**Figure 1:**
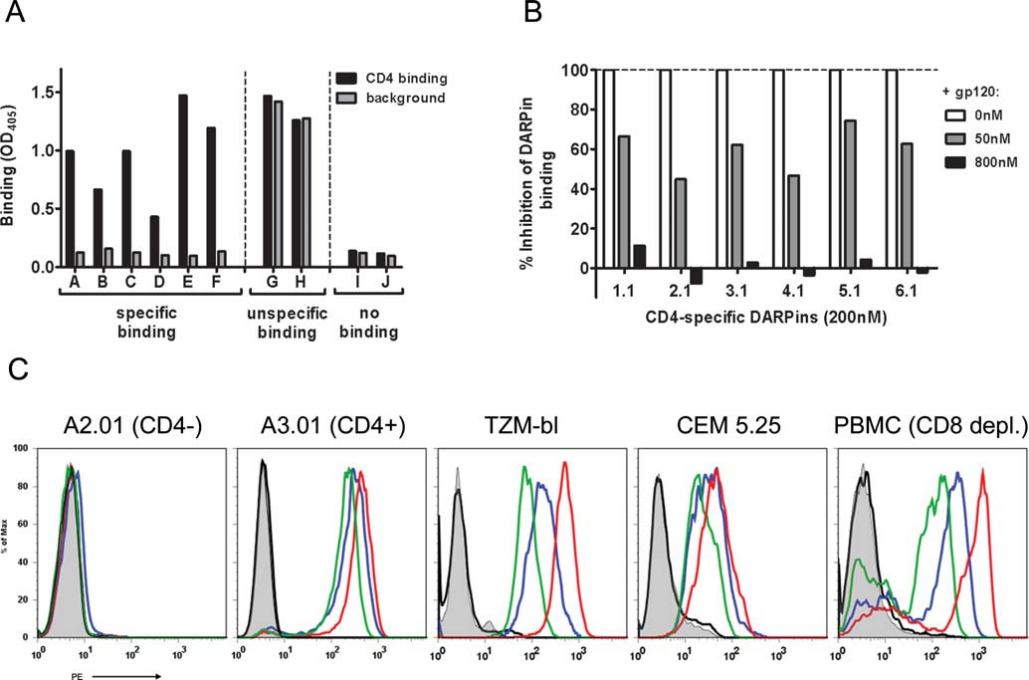
DARPins interact specifically with CD4 and compete with gp120 for binding to CD4. (A) Binding of DARPins, in the form of crude bacterial lysates, to CD4-IgG_2_ (black) is determined by ELISA and compared with binding to the capture antibody alone (gray). DARPins A–F show specific binding to CD4-IgG_2_, a property that was confirmed by further tests using the purified proteins. DARPins G and H reveal nonspecific binding whereas I and J are examples of library members that do not recognize the target protein. (B) Competition ELISA using soluble gp120 as competing ligand. Shown is binding of 200 nM of the CD4-specific DARPins 1.1 to 6.1 in competition with 0 nM, 50 nM and 800 nM gp120. Binding of DARPins alone was defined as 100% and background binding as 0%. (C) Binding of the DARPins to cellular CD4 was tested using A2.01 cells (CD4^−^), the CD4 expressing lines A3.01 cells, TZM-bl cells, CEM 5.25 cells and CD8-depleted PBMC as source of primary CD4 T cells. CD4-specific DARPins D3.1 (blue), D23.2 (red), and of a control DARPin, E3_5 (black), an unselected library member binding to the various cell types are shown. PE-labeled CD4-antibody (clone Q4120, Sigma) (green) was used as positive control and a PE-labeled goat-anti-mouse antibody (shaded in gray) as negative control. The shifts in fluorescence intensity correspond to the differences in affinities of the DARPins for CD4 (see Supporting Table S1). Representative data from 2–4 independent experiments are shown.

DARPins specific for human CD4 were selected using N2C and N3C DARPin libraries, which harbor two and three randomized ankyrin repeats, respectively. Specific DARPins were isolated from these libraries by performing ribosome-display selection rounds [31],[32] against the tetrameric CD4-immunoglobulin molecule, CD4-IgG_2_, expressing domains D1 (encompassing the binding site for the HIV envelope protein gp120; [57]) and D2 of human CD4 [30]. Although an enrichment of binders was observed already after the second ribosome-display selection round (data not shown), four selection rounds were performed to increase specificity before the selected library members were further analyzed. This pool of DARPins obtained after four rounds (referred to as 1^st^ series pool) was screened for CD4 specificity directly from crude bacterial lysates by ELISA (Figure 1A). More than 50% of the examined candidate DARPins showed specific binding (signal/background ≥2), whereas unselected DARPins showed no interaction with immobilized CD4-IgG_2_ (data not shown). Out of this pool of CD4-specific DARPins, six candidate proteins with the most favorable binding properties in the ELISA screen were chosen (referred to as 1^st^ series binders) and purified to homogeneity for further investigations. The six selected proteins were purified and their capacity to bind to CD4 in presence and absence of gp120 assessed (Figure 1B). Notably, all six selected DARPins interfered with gp120 binding to CD4. We further analyzed the ability of DARPins to interact with the native CD4 receptor in a cellular context. All probed selected DARPins bound to CD4^+^ cell lines and to primary CD4^+^ T cells but not to CD4^−^ cell lines, whereas the unselected control DARPin, E3_5, did not interact with any of the tested cell lines (Figure 1C and data not shown).

As affinity and kinetics of the interaction with CD4 are anticipated to steer the efficacy of the DARPins as inhibitors of HIV entry, we investigated the interaction of one candidate from the 1^st^ series pool, DARPin 3.1, with CD4 by kinetic SPR measurements. Association and dissociation experiments at various concentrations of DARPin 3.1 with immobilized CD4-IgG_2_ yielded a dissociation constant (*K_D_* value) of 8.9 nM, which is in the range of high affinity antibodies (Table 1).

**Table 1:** Dissociation constants of CD4 specific DAPRins as determined by surface plasmon resonance (SPR).

Binder	k_on_ 1 [1/Ms]; k_on_ 2 [1/s]	k_off_ 1 [1/s]; k_off_ 2 [1/s]	K_D_ [M]	fitting model	Chi^2^/R_max_
**D3.1**	9.43E+05	1.03E-01	8.93E-09	two state^1^	2.26%
	3.47E−03	3.09E-04			
	**kon [1/Ms]**	**koff [1/s]**			
**D23.2**	2.96E+06	7.66E-04	2.59E-10	1∶1^2^	3.40%
**D27.2**	1.39E+06	2.44E-03	1.75E-09	1∶1^2^	0.48%
**D29.2**	1.11E+06	1.66E-03	1.49E-09	1∶1^2^	4.70%
**D55.2**	1.43E+06	1.20E-03	8.39E-10	1∶1^2^	1.64%
**D57.2**	1.39E+06	2.44E-03	1.75E-09	1∶1^2^	0.48%

1 Data required use of the two state model, which assumes a conformational change upon binding, for fitting.

2 Data gave best fits using the standard 1∶1 langmuir binding model.

### CD4-specific DARPins are potent HIV entry inhibitors

**Figure 2:**
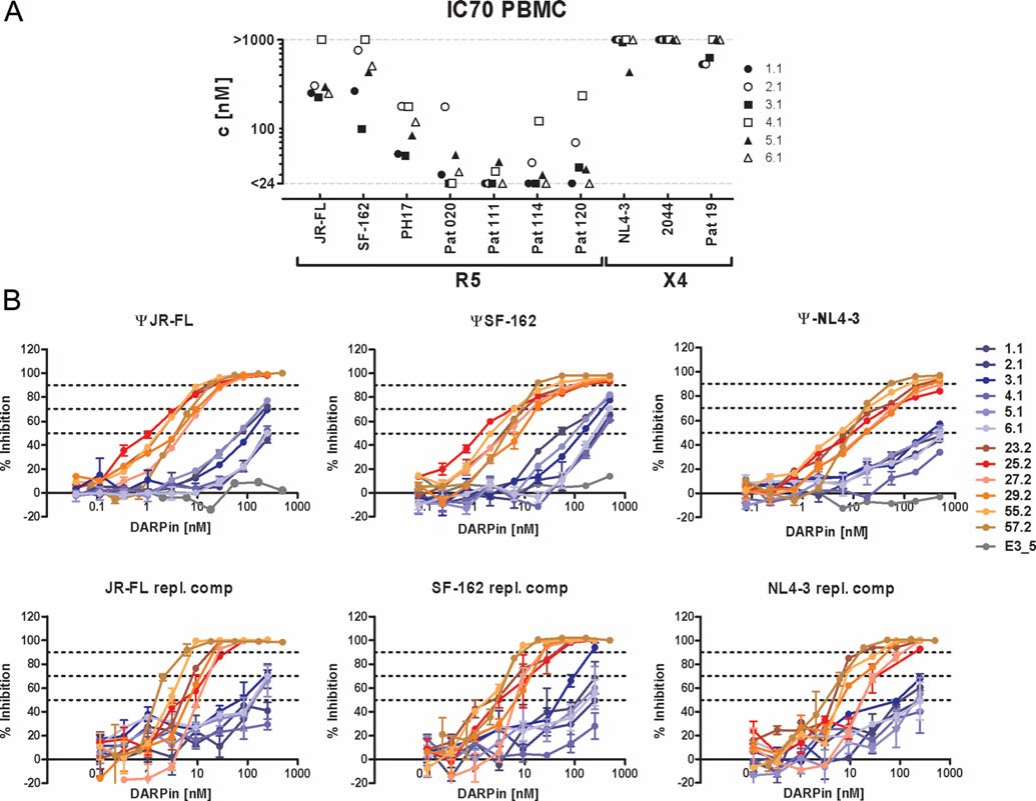
The selected CD4-specific DARPins potently inhibit HIV entry. (A) Effect of the six selected CD4-specific DARPins of the 1^st^ series (D1.1–D6.1) on the entry of replication-competent viruses (7 R5 and 3 X4 users) on PBMC. 70% inhibitory concentrations (IC70) derived in representative individual experiments are reported. (B) Inhibition of HIV entry by 1^st^ and 2^nd^ series DARPins: CD4-specific DARPins of the 2^nd^ series (red to yellow) are more potent inhibitors of HIV entry as DARPins of the 1^st^ series (dark blue to light blue). Inhibition of JR-FL, SF-162 and NL4-3 infection in a pseudotyped virus entry assay on TZM-bl cells (upper panels) and the respective replication competent viruses in a PBMC based assay (lower panels) was probed in parallel. The unselected DARPin E3_5 (gray) was used as control in the TZM-bl based assay. Data points are means of virus replication measured in two replicate wells. See Supporting Table S1 for a summary of the derived IC50 and IC70 values in these assays.

To explore the effect of CD4-specific DARPins on HIV entry we evaluated the inhibitory activity of our panel of CD4-DARPins *in vitro* using a standardized assay system based on infection of TZM-bl reporter cells with envelope pseudotyped HIV particles [58]. All tested DARPins inhibited HIV entry of JR-FL, SF-162 and NL4-3 env-pseudotype viruses in a dose-dependent manner with IC50 values ranging from 67.8 nM to 820 nM (Supporting Table S1). Importantly, none of the CD4-selected DARPins had an effect on CD4-independent virus entry as demonstrated by their inability to block entry of murine leukemia virus (data not shown). Equally, an unselected DARPin (E3_5) had no effect on HIV entry (Figure 2B and data not shown).

When we further explored the effects of the DARPins against a panel of 10 replication-competent R5 or X4 virus isolates of subtype B on primary lymphocytes (Figure 2A) we confirmed that all selected DARPins inhibited replication of the tested virus isolates, even over multiple rounds of replication. Notably though, we observed a considerable variability in the sensitivity of different viruses with IC70 values ranging from <24 nM up to >1 µM, with a relatively high resistance of the three probed X4 isolates to the DARPin inhibitors.

### Selection for improved affinities results in higher inhibition potencies

This relatively high variability in suppressing virus replication on primary CD4^+^ T cells suggested that DARPins with superior activity are needed to achieve potent and broad inhibition of genetically diverse isolates *in vivo*. We reasoned that increasing the affinity of the DARPins to CD4 is the most feasible strategy to boost their potency in inhibiting HIV entry. To enrich for DARPins with high affinity for CD4 we performed off-rate selections during ribosome display to specifically select for proteins with low dissociation rates [59]. To that end, we combined the DNA-sublibraries generated during the first selection rounds and performed a single round of off-rate selection where dissociation of DARPins with low affinity was induced by addition of excess CD4 in solution. From this pool of binders we chose a panel of six DARPins, D23.2, D25.2, D27.2, D29.2, D55.2 and D57.2 (referred to as 2^nd^ series binders), for further analysis.

When we assessed this panel of 2^nd^ series binders using kinetic SPR measurements we found that off-rate selection had indeed resulted in selection of binders with dissociation constants (*K_D_*) that were almost exclusively in the subnanomolar range (Table 1). When compared to DARPin 3.1, the most potent inhibitor of the 1^st^ series, this represents a 5 to 10-fold decrease in *K_D_* values. Importantly, this substantial increase in affinity was also reflected by a dramatic increase in HIV entry inhibition potency of the 2^nd^ series over the 1^st^ series binders (Figure 2B). The IC50 values of the six affinity improved binders against the reference strains JR-FL, SF162 and NL4-3 in the TZM-bl based assay were in the range of 1.1 to 5.1 nM, 1.2 to 7.7 nM, and 2.7 to 10.5 nM, respectively (Supporting Table S1). In sum, this represents about a 70-fold reduction in inhibitory concentrations (p<0.0001, unpaired t test) over the 1^st^ series DARPin inhibitors and renders the 2^nd^ series inhibitors equal in potency to the clinically approved entry inhibitor T-20 [60]–[62], which was probed alongside and inhibited replication of JR-FL, SF-162 and NL4-3 pseudotyped viruses with IC50 values of 1.1 nM, 3.1 nM and 8.1 nM, respectively.

While the 1^st^ series DARPins displayed a relatively wide variability in their potency to inhibit infection of PBMC by replication-competent viruses (Figure 2A, Table S1), the 2^nd^ series DARPins were significantly improved and blocked virus replication at IC70s in the very low nanomolar range (2.1 nM-30.9 nM; Figure 2B and Table S1). The most potent inhibitors of this pool, DARPins 55.2 and 57.2 blocked HIV replication of the three probed viruses, JR-FL, SF-162 and NL4-3, with IC70 values between 2.1 and 7.8 nM.

### Potency and breadth of CD4-specific DARPins

**Figure 3:**
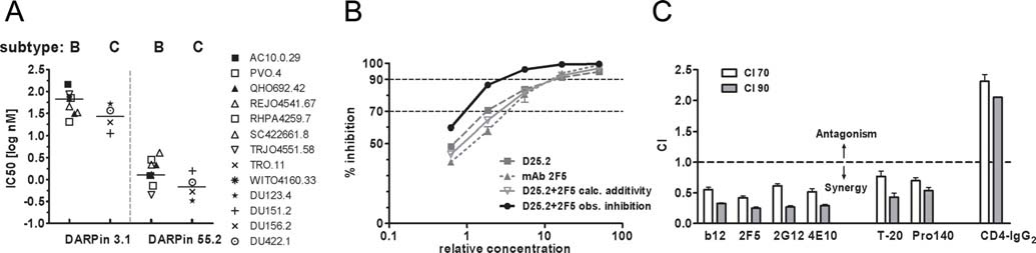
CD4-specific DARPins efficiently inhibit entry of both subtype B and C viruses. (A) Graphical representation of the IC50 values of a 1^st^ series DARPin, D3.1, and a 2^nd^ series DARPin, D55.2, tested using env-pseudotyped viruses of subtype B (n = 9) and subtype C (n = 4) on TZM-bl cells. The following median IC50 values were determined: 45.4 nM for DARPin 3.1 (67.1 nM and 28.2 nM for clade B and C viruses, respectively) and 1.3 nM for DARPin 55.2 (1.3 nM and 0.7 nM for clade B and C viruses, respectively). (B) Inhibition curves of JR-FL pseudovirus used to assess synergy by analyzing combination indices (CI) in Figure 3C. Equipotent stocks of inhibitors were used to obtain comparable inhibition curves. Inhibitory effects of DARPin 25.2 (gray square) and the 2F5 mAb (gray triangle) alone are shown aside by side with the calculated (light gray, open triangle) and the actual observed inhibitory effect (black circles) of a 1∶1 mixture of the two inhibitor stocks. (C) DARPin 25.2 shows potent synergy in JR-FL pseudovirus inhibition in combination with neutralizing mAbs or entry inhibitors. CI for the inhibitory concentrations 70% and 90% (CI70, CI90) are represented for DARPin 25.2 in combination with mAbs IgG-b12, 2F5, 2G12, 4E10, the fusion inhibitor T-20, the anti-CCR5 mAb Pro140 and CD4-IgG_2_. Means from three independent experiments are shown. Error bars indicate the standard error of the mean.

To obtain more detailed information on potency and breadth of the CD4-specific DARPins we analyzed the activity of DARPin 3.1, the most potent inhibitor of the 1^st^ series pool, and DARPin 55.2, as representative of the 2^nd^ series, against a reference panel of nine subtype B and four subtype C *env*-pseudotyped R5 viruses (Figure 3A). Notably, D3.1 only reached IC50 values between 20.2 and 144.8 nM (median: 67 nM) against clade B viruses and 11.3 to 52.5 nM (median: 28 nM) against clade C viruses while DARPin 55.2 inhibited both subtype B and C viruses very potently with IC50 values of 0.4–4.1 nM for subtype B (median: 1.3 nM) and 0.3–1.6 nM for subtype C viruses (median: 0.7 nM). The latter confirmed the result of the initial screen and verified that the 2^nd^ series DARPins have a markedly improved capacity to inhibit HIV, irrespective of the genetic background of the virus.

As with all inhibitors against HIV, effective application of CD4-specific DARPins for prevention or therapy will require their use in combination with other types of inhibitors. To probe potential effects of CD4-DARPins in drug cocktails, DARPin 25.2 was tested for its efficacy in inhibiting HIV entry in combination with a series of entry inhibitors: the neutralizing mAbs IgG-b12 [63], 2F5 [64], 4E10 [65] and 2G12 [66], the fusion inhibitor T-20 [60], the anti-CCR5-mAb PRO140 [67] and CD4-IgG_2_ [30].

The results showed a clear pattern: DARPin 25.2 acted in synergy (CI 70: 0.42–0.77, CI 90: 0.25–0.54) with all anti-cell and anti-viral inhibitors with the exception of CD4-IgG_2_ for which - consistent with the CD4-specificity of the DARPins - antagonism was observed (CI 70: 2.31, CI 90: 2.05; Figure 3B and C). The precise mechanisms by which blocking of CD4 promotes synergistic effects in combination with anti-envelope targeting inhibitors remain to be determined. Synergistic effects could, for example, arise when thresholds of receptor levels required for successful entry are not met. In summary, our data underline the potential of CD4-specific DARPin inhibitors, as they promote higher inhibitory activity in conjunction with entry inhibitors directed to different targets.

### Specificity of selected DARPins

To derive further information on their target specificity, we studied binding of a selection of 2^nd^ series DARPins to CD4 in competition with a panel of CD4-binding mAbs. In general, strong competition with the three D1 binding mAbs (L222, Q4120, 13B82 [34],[35]) was observed, while less interference was found with 5A8 [36], a D2 binding antibody (Table 2). Notably, this competition by mAb 5A8 was not observed with DARPin 23.2, but with all other tested DARPins.

**Table 2:** Competition between DARPins and CD4-specific antibodies for binding to CD4.

mAb/DARPin	−	E3_5	23.2	27.2	29.2	55.2	57.2
**−**	**−**	**−**	**−**	**−**	**−**	**−**	**−**
**5A8**	**−**	**−**	**−**	**+**	**++**	**+**	**+**
**L222**	**−**	**−**	**+++**	**+++**	**+++**	**++**	**++**
**Q4120**	**−**	**−**	**+++**	**+++**	**+++**	**+++**	**++**
**13B82**	**−**	**−**	**++**	**+**	**++**	**++**	**+**
**α-Flag**	**−**	**−**	**−**	**−**	**−**	**−**	**−**

**Figure 4:**
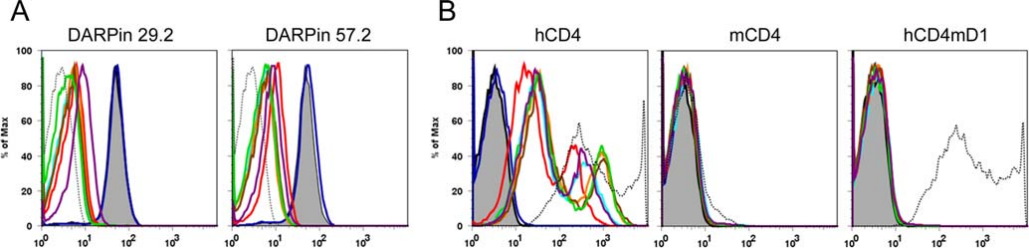
Characterization of the binding domain of CD4 specific DARPins. (A) Competition between the fluorescently labeled DARPins D29.2HLX and D57.2HLX with unlabeled DARPins was analyzed by flow cytometry using CD4^+^ A3.01 cells. Compared to the control with no competitor (shaded in gray) competitive binding was observed for all CD4-specific DARPins (E3_5: blue; D23.2: light blue; D25.2 red; D27.2: orange; D29.2: brown; D55.2: green; D57.2: purple). The autofluorescence control is shown as dotted line (B) Binding of the same DARPins to human CD4 (hCD), murine CD4 (mCD4) and chimeric human CD4 containing murine D1 domain in the human CD4 context (hCD4mD1), indicating that all tested DARPins bind to the D1 domain of human CD4. The same coloring scheme for DARPins as in Figure 4A was used. MAb OKT4, specific for human CD4-D3, is shown as black dotted line.

In summary these experiments suggest that the selected DARPins have overlapping specificities mainly directed against D1. We confirmed these experiments in competition experiments in which binding of fluorescently labeled DARPin 29.2 or 57.2 to CD4 expressing cells was probed in presence of unlabeled competitor DARPins (Figure 4A). Both sets of experiments gave identical results: the labeled DARPin was competed off by all other CD4 specific but not the control DARPin E3_5, indicating that the probed CD4-specific DARPins have closely overlapping epitopes.

To more specifically define the binding domain of the DARPins we generated a chimeric CD4 molecule in which domain 1 of human CD4 was exchanged by the corresponding domain of mouse CD4. The chimeric CD4 molecule expressed well upon transfection in 293-T cells, and had the required specificities, as antibody S3.5, specific for human D1, failed to bind, whereas mAb GK1.5, specific for mouse D1, bound the chimeric molecule but not wild type human CD4 (data not shown). Likewise mAb OKT4, specific for human CD4 D3, bound equally well to both wildtype human CD4 and the chimeric molecule (Figure 4B). Binding studies with the CD4 specific DARPins revealed that while all DARPins bound wildtype human CD4, they failed to bind the chimeric mouse domain 1 molecule mirroring the binding pattern of mAb S3.5 and thus confirming their specificity for CD4 domain 1 (Figure 4B).

### Probing the effect of CD4-specific DARPins on cell function

**Figure 5:**
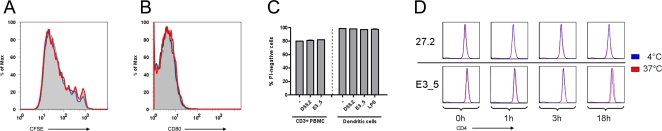
Interaction of DARPins with CD4 has no detectable effect on cell viability and stimulation. (A) PBMC stimulation with IL-2 and OKT3 to induce proliferation was not altered in presence of the CD4-specific DARPin 55.2 (red), the non-binding DARPin E3_5 (blue) or absence of DARPin (gray) over a 4 day period. Proliferation was monitored by flow cytometry by determining CFSE dilution as a result of cell division. One representative experiment out of two is shown. (B) Activation of dendritic cells (DC) as determined by CD80 expression. Neither addition of DARPin 55.2 (red), nor of control DARPin E3_5 (blue), resulted in detectable upregulation of CD80 on DC over a 24 h period. One out of two independent experiments is depicted. (C) Prolonged treatment of T lymphocytes (4 days) and immature monocyte derived DC (24 h) with DARPin 55.2 (CD4 specific) and E3_5 (unselected control DARPin) has no effect on cell viability. Viability was determined by propidium iodide staining. DARPin concentration in the lymphocytes and DC cultures were 500 nM and 375 nM, respectively. (D) Interaction of DARPins with CD4 does not result in downregulation of surface CD4. Untouched peripheral blood CD4^+^ T cells were cultured in presence or absence of the indicated DARPins for 1 h, 3 h or 18 h at either 37°C (red line) or 4°C (blue line). Cells were stained for CD4 and the expression of surface CD4 was analyzed by flow cytometry. Shown is one representative experiment out of four.

Since the action of CD4-specific DARPins is directed against the host cell, particular care has to be taken to assess their effect on cell function before these agents can be considered for further development as HIV inhibitors. In a first step, we investigated whether CD4-specific DARPins interfere with CD4 T cell proliferation, by probing the effect of a candidate CD4-specific DARPin (D55.2) and a nonspecific control DARPin (E3_5) on primary CD4^+^ T cell proliferation over a four day period. As Figure 5A shows, addition of the CD4-binding DARPin had no noticeable impact on cell proliferation compared to the untreated control.

To explore the effects of CD4 engagement by DARPins on dendritic cells (DC), we assessed whether treatment of immature monocyte-derived DC with DARPin 55.2 for 24 h induced activation and maturation of these cells, which is reflected by increased expression of the costimulatory molecule CD80. Neither the CD4-specific DARPin 55.2 nor the control DARPin induced DC maturation (Figure 5B), whereas *E. coli* lipopolysaccharide (LPS), known to induce DC maturation via TLR-4, gave rise to a pronounced shift in CD80 expression (data not shown).

Notably, the DARPins did not reveal any cytotoxic effects: prolonged incubation of primary cells with DARPin - CD4-specific or unselected - did not result in increased cell death as measured by uptake of propidium iodide: Both the CD3+ T cells (incubated with DARPins, 500 nM, for 4 days) and the dendritic cells (incubated with DARPins, 375 nM, for 24 h) remained unaffected (Figure 5C).

### Effect on CD4 receptor density

As our competition binding experiments with gp120 indicate (Figure 1B), CD4-specific DARPins most likely act by blocking viral attachment to the receptor. Theoretically, binding of the DARPin to CD4 could also induce receptor internalization and DARPins thus may exhibit their antiviral activity through decreasing CD4 receptor density on the target cells. To probe this, we explored the effect of DARPin binding on surface CD4 receptor levels of primary CD4 T cells. Treatment of CD4 T cells from healthy donors with DARPin for 0, 1, 3 and 18 h at 37°C (to allow receptor internalization) or at 4°C (to limit internalization) yielded identical results: Neither treatment with the CD4 specific nor the unspecific DARPin resulted in down- or upregulation of CD4 (Figure 5D). Recognition of CD4 by the CD4 mAb used in these FACS analyses was not impaired in the presence of CD4 specific DARPin. Most importantly, CD4 staining in presence of CD4 specific DARPin remained stable independently whether DARPin and mAb were added simultaneously or cells were pretreated with DARPin for extended time periods (Figure 5D).

### Effect of CD4 specific DARPins on CD4 interaction with MHC class II

**Figure 6:**
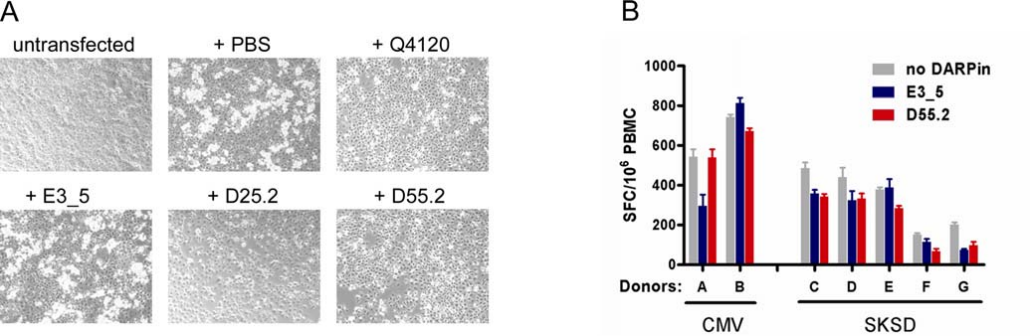
Effect of DARPin on T cell function and MHC class II interaction. (A) The effect of the DARPin∶CD4 interaction was assessed in a binding assay based on rosette formation between CD4 and MHC class II expressing cells. Rosette formation was blocked by all tested CD4 specific DARPins (200 nM, shown are D25.2 and D55.2) or by the CD4-specific mAb Q4120 but not by the control DARPin E3_5. One out of two representative experiments is shown. (B) ELISpot assay to detect IFN-γ production by activated T cells showed no interference of DARPin 55.2 with CD4^+^ T cell activation. The response of two donors against CMV or streptokinase/streptodornase (SKSD) antigen was tested without DARPin (gray) and with nonspecific (blue) or CD4-specific DARPin (red) at 200 or 250 nM. One out of two independent experiments is depicted.

In the absence of T cell receptor interaction the binding of CD4 to MHC class II is of extremely low affinity (*K_D_* = 200 µM; [68]). Using a previously established assay that allows to study this weak interaction based on rosette formation between CD4 and MHC-II expressing cells [56], we were able to show that all tested DARPins, 23.2, 25.2, 29.2, 55.2 and 57.2, as well as the CD4-D1 specific antibody Q4120 blocked rosette formation efficiently (Figure 6A and data not shown). Hence, in the absence of cognate T cell receptor (TCR) and peptide, the CD4 specific DARPins interfered with CD4 binding to MHCII.

To probe the effect on specific T cell functions, we assessed if the CD4-specific DARPin 55.2 affects activation of memory T helper cells specific for either streptokinase/streptodornase or cytomegalovirus antigens. When we quantified antigen specific IFN-γ producing cells that were stimulated in presence or absence of 200 nM of D55.2 or the non-binding control DARPin E3_5 we observed in both cases no inhibition of the T cell functions (Figure 6B). This indicates that, at least for the CD4-specific DARPin probed, even at high dosing of the molecule specific memory T helper responses are activated.

### Efficacy of DARPins in blocking rhesus macaque CD4

**Figure 7:**
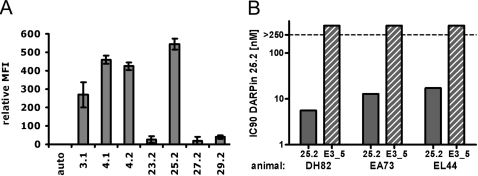
Human CD4 specific DARPins can crossreact with macaque CD4 and inhibit entry of SIV. (A) PBMC from rhesus macaques were incubated with the indicated DARPins at 4°C, labeled with an anti-His-tag antibody and detected by flow cytometry. MFI (mean fluorescence intensity) of DARPin staining on CD3^+^ cells, corrected for the negative control is shown. Standard deviations are indicated and represent n = 4 animals. (B) DARPin 25.2 potently inhibits entry of SIVmac239 in macaque PBMC. Shown are the IC90 values which were derived from neutralization assays performed using DARPin 25.2 and the control E3_5 on PBMC from three different animals.

To evaluate the potential of using these binders directly in non-human models, crossreactivity of the DARPins with CD4 from rhesus macaques was investigated. The sequence identity between human and macaque CD4 is 91% on the amino acid level, as opposed to 54% between human and murine CD4. Experiments using PBMC from macaques revealed that 4 out of 7 tested DARPins recognize also rhesus CD4 (Figure 7A), while none of them interacts with murine CD4 (data not shown and Figure 4B). This finding is intriguing as it opens the possibility to probe the potential of DARPins as candidate microbicides in the macaque infection model. To obtain an initial insight into the potential of these DARPins in inhibiting SIV infection, we probed the efficacy of DARPin 25.2 in blocking SIVmac239 infection of primary rhesus macaque cells. Results obtained in infection experiments with cells from three individual donors depicted in Figure 7B indicate that DARPin 25.2 potently inhibit SIV infection of these cells.

## Discussion

Making use of the recently developed DARPin technology [19]–[21],[23], we investigated here DARPins as HIV-specific inhibitors since they can be engineered to fulfill many of the sought for properties of a microbicide, namely high target specificity and affinity, high physical stability and comparatively low production costs. As proof of concept, we aimed to derive DARPin-based inhibitors that target CD4, the primary receptor for HIV. The technology employs highly diverse DARPin DNA libraries combined with ribosome display as selection technology, which allowed the selection of binders with specificity for the CD4 receptor in a relatively short time. The resulting DARPins interacted with very high affinity with human CD4 as reflected by dissociation constants in the lower nanomolar range, which upon off-rate selection even reached subnanomolar values. This high affinity has proven a common characteristic of DARPins: although monovalent binders, they routinely achieve affinities that are equal if not superior to most antibodies [23],[26],[28],[59]. We subjected the derived CD4-specific DARPins to a careful assessment of the HIV inhibitory capacity. Notably, all probed CD4-specific DARPins from the 1^st^ and the 2^nd^, affinity improved series inhibited HIV entry both in cell line and primary cell based infection systems. Inhibition was achieved over both single round and multiple rounds of infection proving the stability of this effect. Particularly notable was the potency of the 2^nd^ series DARPins, which were specifically selected for low dissociation rates. They exhibited potent and broad neutralization of HIV across subtypes B and C at IC50 values in the low nanomolar range. The latter proved them to be at least equally potent as the licensed entry inhibitor T-20 (Enfuvirtide; [69],[70]).

We used a truncated CD4 molecule, expressing only the apical D1 and the adjacent D2 domain of CD4 as target in the ribosome display selection, as these domains are involved in the interaction with the virus and targeting of these regions by specific antibodies have been shown to interfere with HIV infection [71]–[75]. D1 harbors the binding site for gp120 and interference is expected to abrogate this interaction. The role of D2 in the infection process appears to be more indirect, nevertheless important: the D2 specific antibody 5A8 blocks HIV infection efficiently and its humanized derivative TNX-355 is now under clinical investigation [69],[76],[77]. Notably, all tested DARPins selected against D1 and D2 of CD4 in our screen inhibited HIV entry. The most obvious concept of inhibiting HIV entry is blocking of the gp120 binding site within the D1 domain of CD4 and thus direct interference with viral attachment. Competition between virus and inhibitor could likewise arise from binding to an epitope on D1 that is different from the gp120 binding site, resulting in either conformational changes or in stabilization of an incompatible conformation of the entire domain.

Our screening strategy should enrich for DARPins specific for D1, as competitive displacement from CD4 by gp120 was applied in the final ribosome display rounds. It also has to be considered that the D2 domain is probably less exposed in the tetrameric form of CD4-IgG_2_ and therefore likely not as accessible for DARPin binding during the screening. More detailed epitope mapping using mouse human CD4 chimera showed that indeed all selected DARPins bind to domain 1 of human CD4.

Notably though, we observed a partial competition between the D2 specific mAb 5A8 and several of the DARPins for CD4 binding, indicating that the epitopes of these DARPins may also involve regions in D2 or are dependent on a D2 steered conformation.

We further found that engagement of CD4 by the DARPins 27.2, 29.2, 55.2, and 57.2 did not induce downregulation of CD4 (Figure 5D and data not shown), supporting the notion that direct interference with gp120 binding to CD4 is their mode of action (Figure 1B).

Although we developed the DARPin inhibitors with topical application as a microbicide in mind, where comparatively low systemic exposure is expected, it is nevertheless critical to carefully assess their potential side effects on immune function. Despite targeting a cellular receptor, we found the actions of the selected CD4-specific DARPins to be highly HIV specific. No effect on CD4-independent virus entry was detected using murine leukemia virus. Equally important, we did not observe effects on cell viability, proliferation of T-cells, or activation of immature DC for the individual DARPins probed in these assay systems, indicating that these monovalent binders did not activate the receptor and initiated downstream signaling events. Moreover, although DARPins can interfere with the low affinity interaction between CD4 and MHC class II which occurs in the absence of cognate TCR and peptide (Figure 6A; [68]), DARPin treatment did not disturb activation of specific memory T helper responses (Figure 6B). The latter supports previous observations that CD4/MHC class II interaction is tightened on TCR engagement [68],[78], which may explain why the inhibitory effect of the DARPin is overcome in this context.

The fact that targeting of CD4 by the high affinity DARPins can occur without loss of CD4 T cell function and unwanted side effects, holds great promise of their *in vivo* application. This is further underlined by our finding that CD4-specific DARPins act in synergy with several other HIV entry inhibitors directed to different targets on the virus or host cell. The DARPin technology is a relative young invention and the potential *in vivo* applications of DARPins still await proof. This notwithstanding, our *in vitro* analysis strongly suggests that DARPins have unique properties that render them promising candidates for microbicide development. Further assessment of their application as microbicides is clearly feasible, particularly as we selected several molecules that are specific for human and rhesus macaque CD4, which will allow future study of their efficacy in the macaque infection model.

### Accession numbers

The nucleotide and the amino acid sequences of the 12 DARPins described here were deposited in the EMBL Nucleotide Sequence Data Base (www.ebi.ac.uk/embl) and are available under the accession numbers AM997259–AM997270.

## Supporting Information

Protocol S1 Designed Ankyrin Repeat Proteins (DARPins) and ribosome display.
(0.07 MB DOC)

Figure S1 Repeat sequence motif of a DARPin repeat and X-ray structure of a randomly selected member of the N3C DARPin library, E3_5.
(0.82 MB TIF)

Figure S2 Schematic representation of ribosome display selections.
(0.10 MB TIF)

Table S1 Inhibitory concentrations of 1st and 2nd series DARPins.
(0.07 MB DOC)
