# Supplementary material for: CD4-Specific Designed Ankyrin Repeat Proteins Are Novel Potent HIV Entry Inhibitors with Unique Characteristics
Source: PLoS Pathog. 2008 Jul 25;4(7):e1000109. doi: 10.1371/journal.ppat.1000109 (PMC2453315; doi:10.1371/journal.ppat.1000109)
Supplement: Protocol S1 — Designed Ankyrin Repeat Proteins (DARPins) and ribosome display. (0.07 MB DOC) [file ppat.1000109.s001.doc]

### **Protocol S1**

### **Designed Ankyrin Repeat Proteins (DARPins)**

The concept to generate combinatorial libraries of Designed Ankyrin Repeat Proteins (DARPins) was developed by Andreas Plückthun and coworkers, exploiting the favorable characteristics of repeat proteins [1,2]. An extensive review on this method has been recently published by Forrer *et al.* [3]. Ankyrin repeat proteins form one of the largest protein families in nature and feature a highly conserved sequence and structure. Plückthun and colleagues derived a con­sensus Ankyrin sequence for the development of self-compatible repeat modules, termed DARPins. As shown in Figure S1, the architecture of solenoid repeat proteins such as the DARPins can be dissected into two dissimilar parts:

- Internal repeats, which form the largest, elongated part of the domain. These internal repeats, comprising constant framework residues as well as randomized amino acids, were designed applying the concept of library design [2].
- The constant N- and C-terminal capping repeats that shield the hydrophobic core of the proteins from the aqueous solvent and are hence relevant for the stability and the solubility of these proteins [2].

The final consensus sequence of the 33 amino acids of the internal DARPin repeat was set as: DxxGxTPLHLAAxxGHLEIVEVLLKZGADVNAx, where x can be any amino acid except Cys, Pro or Gly and Z stands for His, Asp or Tyr. Depending on the number of repeats, DARPins are classified as N1C, N2C, N3C etc., where N and C stand for the N- and C-terminal capping repeats and the number indicates how many variable internal AR modules are present. With seven randomized positions per designed ankyrin repeat unit, the theoretical diversity amounts to 7.2 x 10^7^ per repeat, while the N2C and the N3C library will thus have theoretical diversities of 5.2 x 10^15^ and 3.8 x 10^23^, respectively [2]. Individual members of these very diverse libraries can then be selected for binding to any given target protein using a display technology such as ribosome display [4].

### **Ribosome Display**

DARPins were selected using ribosome display [5,6], a system which provides physical linking of the individual proteins or peptides, the phenotype, with their genetic bluescript, the genotype. The fundamental feature of ribosome display is the formation of ternary protein-ribosome-mRNA complexes. A detailed description of this methodology can be found at Zahnd *et al.* [6]. Ribosome display is a cell-free system, relying on the non-covalent coupling of mRNA to the nascent protein, for the *in vitro* selection of proteins and peptides from large libraries [5]. As shown in the figure below, the DARPin cDNA library is transcribed into mRNA using recombinant RNA polymerase. The resulting mRNA library is then translated *in vitro*, using crude *E. coli* extract, which contains all components required for translation, including ribosomes and tRNAs. Additional components such as ATP are also added to ensure efficient translation. It is crucial for the selection procedure that the mRNA genotype is coupled to the protein phenotype via the ribosome. To that end, stop codons were removed from the DARPin library and the *in vitro* translation is carried out under high Mg^2+^ concentrations at 37°C for only 5-10 minutes. The ternary mRNA-ribosome-DARPin complexes are stable at 4°C and can be tested for binding DARPin component, to an immobilized target, here a variant of CD4. The very large size of the DARPin library ensures that some proteins will bind the target with reasonable affinity, allowing all others to be removed by the sub­sequent washing step. To obtain the genetic information of proteins that recognize the target protein, the ternary complexes are disassembled using EDTA and the mRNA is recovered using standard molecular biology protocols. Finally, the DNA is recovered via reverse transcription, thereby completing the first cycle of ribosome display [1,7,8]. Three to four rounds of ribosome display, typically with more intense washing from round to round, are required to sufficiently enrich proteins that bind their targets specifically and with high affinity (Figure S2).

The selected library members can then be tested for binding using methods as ELISA and surface plasmon resonance (SPR) analysis [1,9] or their properties can be analyzed in cell-based assays. Compared to other selection techniques ribosome display is particularly well suited for very large libraries such as DARPin libraries since no transformation steps limit the applicable library size. Furthermore, the fact that living cells are not required enables the selection of cytotoxic proteins or of proteins with limited *in vivo* stability [9].

Off-rate selection is a variation of ribosome display that selects specifically for low off-rates of the DARPin-target protein. It basically relies on the formation of complexes consisting of the ternary mRNA-Ribosome-DARPin entities bound to biotin-labeled target protein. An excess of unlabeled target protein is added and after a few hours the labeled complexes are pulled down using streptavidin coated magnetic particles [10].

**Figure Legends:**

**Figure S1.** **Repeat sequence motif of a DARPin repeat and X-ray structure of a randomly selected member of the N3C DARPin lib­rary, E3_5.** A) The designed DARPin sequence motif. Randomized positions are indi­cated with X (any amino acid except Cys, Gly, Pro) and Z (His, Asn, Tyr), constant residues are in black. D) Ribbon representation of E3_5 [11], displaying the N- and the C-terminal capping re­peats in light gray and the internal repeats in light red, re­spectively. Side chains of variable amino acids are displayed in stick representation and highlighted in red. The figure was generated using the program Pymol ([www.pymol.org](http://www.pymol.org/))

**Figure S2. Schematic representation of ribosome display selection.** The selection system relies on the coupling of correctly folded proteins, the phenotype, with their encoding genetic material, the genotype. In contrast to other display technologies, ribosome display does not contain any transformation steps and is thus a complete *in vitro* technology.

**References**

1. Binz HK, Amstutz P, Kohl A, Stumpp MT, Briand C, et al. (2004) High-affinity binders selected from designed ankyrin repeat protein libraries. Nat Biotechnol 22: 575-582.

2. Binz HK, Stumpp MT, Forrer P, Amstutz P, Plückthun A (2003) Designing repeat proteins: well-expressed, soluble and stable proteins from combinatorial libraries of consensus ankyrin repeat proteins. J Mol Biol 332: 489-503.

3. Forrer P, Binz HK, Stumpp MT, Plückthun A (2004) Consensus design of repeat proteins. ChemBioChem 5: 183-189.

4. Stumpp MT, Amstutz P (2007) DARPins: a true alternative to antibodies. Curr Opin Drug Discov Devel 10: 153-159.

5. Hanes J, Plückthun A (1997) In vitro selection and evolution of functional proteins by using ribosome display. Proc Natl Acad Sci U S A 94: 4937-4942.

6. Zahnd C, Amstutz P, Plückthun A (2007) Ribosome display: selecting and evolving proteins in vitro that specifically bind to a target. Nat Methods 4: 269-279.

7. Amstutz P, Binz HK, Zahnd C, Plückthun A (2004) Ribosome display: In vitro selections of protein-protein interactions. in press.

8. Schaffitzel C, Plückthun A (2001) Protein-fold evolution in the test tube. Trends Biochem Sci 26: 577-579.

9. Amstutz P, Forrer P, Zahnd C, Plückthun A (2001) In vitro display technologies: novel developments and applications. Curr Opin Biotechnol 12: 400-405.

10. Zahnd C, Pecorari F, Straumann N, Wyler E, Pluckthun A (2006) Selection and characterization of Her2 binding-designed ankyrin repeat proteins. J Biol Chem 281: 35167-35175.

11. Kohl A, Binz HK, Forrer P, Stumpp MT, Plückthun A, et al. (2003) Designed to be stable: crystal structure of a consensus ankyrin repeat protein. Proc Natl Acad Sci U S A 100: 1700-1705.
