# Supplementary figures and images for: CD4-Specific Designed Ankyrin Repeat Proteins Are Novel Potent HIV Entry Inhibitors with Unique Characteristics
Source: PLoS Pathog. 2008 Jul 25;4(7):e1000109. doi: 10.1371/journal.ppat.1000109 (PMC2453315; doi:10.1371/journal.ppat.1000109)

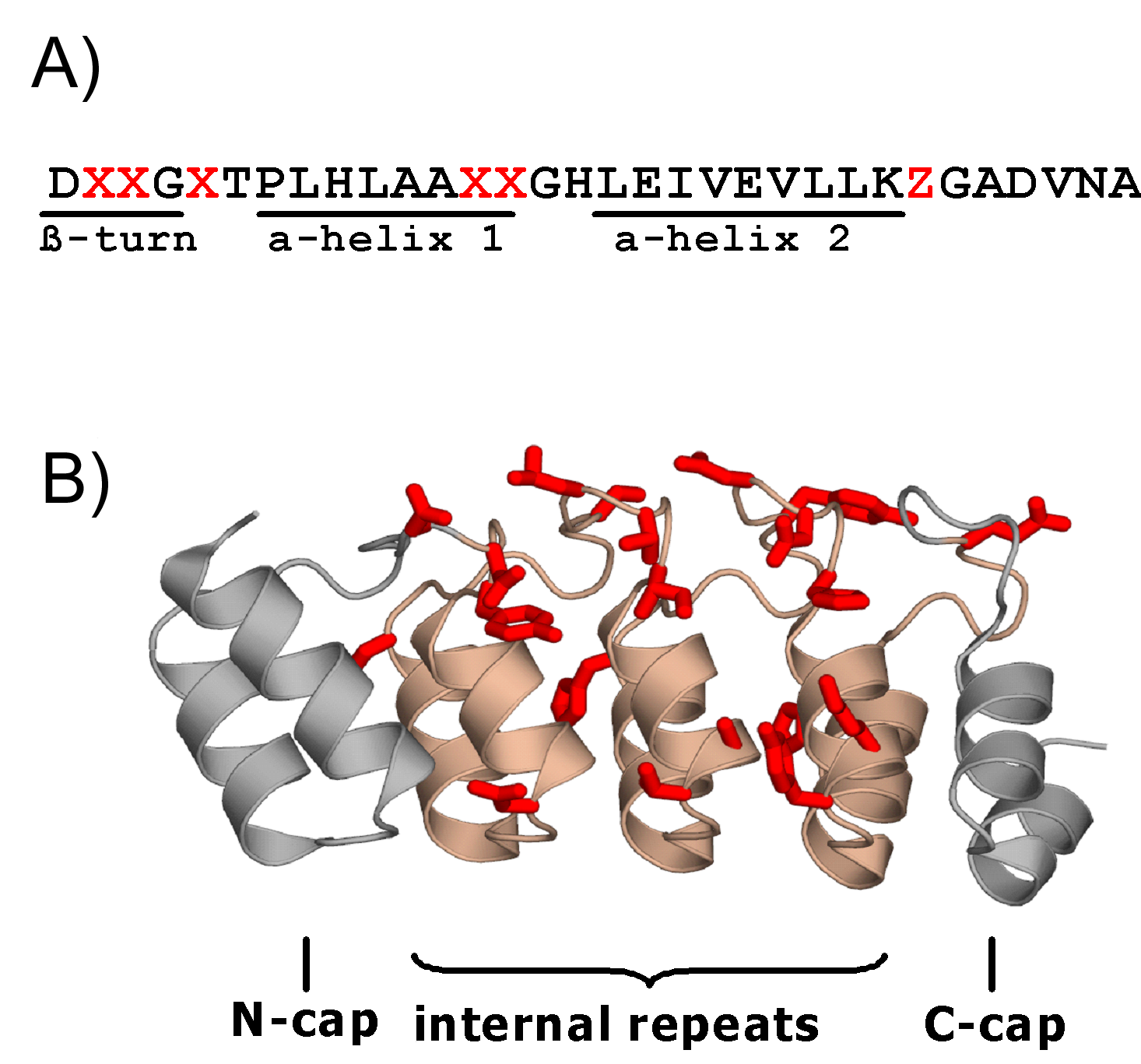

Supplement: Figure S1 — Repeat sequence motif of a DARPin repeat and X-ray structure of a randomly selected member of the N3C DARPin library, E3_5. (0.82 MB TIF) [file ppat.1000109.s002.tif]

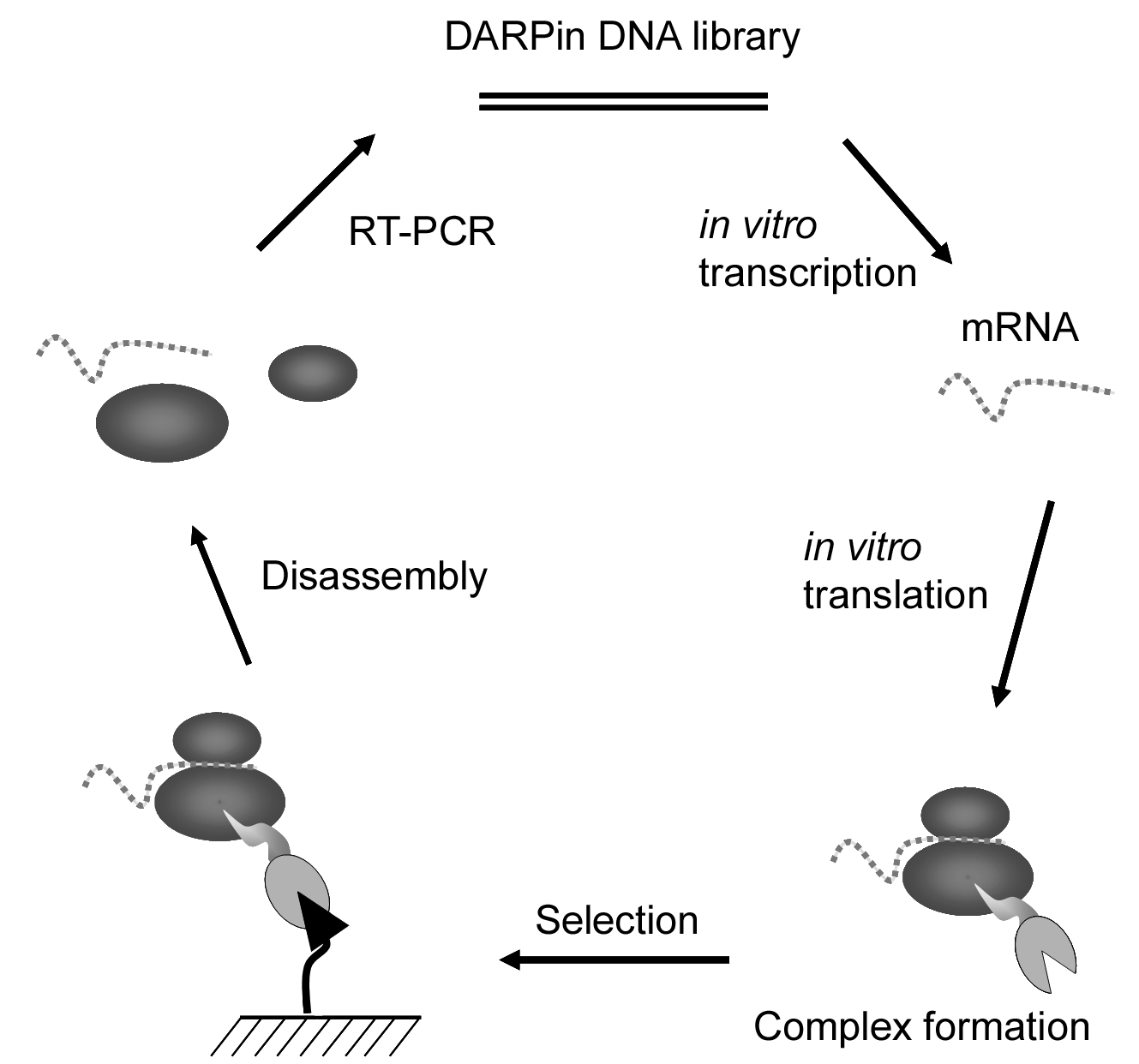

Supplement: Figure S2 — Schematic representation of ribosome display selections. (0.10 MB TIF) [file ppat.1000109.s003.tif]
