# Supplementary material for: CD4-Specific Designed Ankyrin Repeat Proteins Are Novel Potent HIV Entry Inhibitors with Unique Characteristics
Source: PLoS Pathog. 2008 Jul 25;4(7):e1000109. doi: 10.1371/journal.ppat.1000109 (PMC2453315; doi:10.1371/journal.ppat.1000109)
Supplement: Table S1 — Inhibitory concentrations of 1st and 2nd series DARPins. (0.07 MB DOC) [file ppat.1000109.s004.doc]

| **Table S1:** Inhibitory concentrations of 1^st^ and 2^nd^ series DARPins | | | | | | | |
| --- | --- | --- | --- | --- | --- | --- | --- |
|  | **pseudotyped** | | |  | **repl.-competent** | | |
|  |  |  |  |  |  |  |  |
| Virus | **JR-FL** | **SF-162** | **NL4-3** |  | **JR-FL** | **SF-162** | **NL4-3** |
| Inhibitory concentration | **IC_50_** | **IC_50_** | **IC_50_** |  | **IC_70_** | **IC_70_** | **IC_70_** |
|  |  |  |  |  |  |  |  |
| **1^st^ series DARPins** |  |  |  |  |  |  |  |
| **D1.1** | 72 | 67 | 738 |  | 250 | 264 | >1000 |
| **D2.1** | 315 | 273 | 820 |  | 304 | 762 | >1000 |
| **D3.1** | 112 | 132 | 292 |  | 226 | 99 | 937 |
| **D4.1** | 229 | 310 | 773 |  | >1000 | >1000 | >1000 |
| **D5.1** | 68 | 89 | 202 |  | 296 | 436 | 432 |
| **D6.1** | 253 | 227 | 570 |  | 250 | 510 | >1000 |
|  |  |  |  |  |  |  |  |
|  |  |  |  |  |  |  |  |
| **2^nd^ series DARPins** |  |  |  |  |  |  |  |
| **D23.2** | 1.8 | 3.4 | 3.7 |  | 7.9 | 6.9 | 5.9 |
| **D25.2** | 1.1 | 1.2 | 4.4 |  | 15.0 | 12.5 | 22.6 |
| **D27.2** | 5.1 | 4.2 | 10.5 |  | 16.3 | 9.3 | 30.9 |
| **D29.2** | 3.9 | 7.7 | 8.4 |  | 11.5 | 13.0 | 16.4 |
| **D55.2** | 1.7 | 2.0 | 2.7 |  | 4.6 | 4.3 | 7.8 |
| **D57.2** | 3.7 | 4.3 | 8.7 |  | 2.1 | 4.4 | 6.8 |
|  |  |  |  |  |  |  |  |
